# Clinical efficacy of ERAS plus enteral nutrition in patients undergoing laparoscopic surgery for gynecologic malignancies

**DOI:** 10.3389/fmed.2025.1576761

**Published:** 2025-10-03

**Authors:** Huan Xia, Yaoyang Zhang, Cuiying Su, Min Zhou, Wenying Liu, Jiming Chen, Bingying Lu

**Affiliations:** ^1^Department of Obstetrics and Gynecology, Zigong Fourth People’s Hospital, Zigong, Sichuan, China; ^2^Department of Obstetrics and Gynecology, The Second Affiliated Hospital of Dalian Medical University, Dalian, Liaoning, China; ^3^Department of Gynecology and Obstetrics, The Affiliated Changzhou Second People’s Hospital of Nanjing Medical University, Changzhou, Guangdong, China

**Keywords:** ERAS, enteral nutrition, gynecological malignant tumor, laparoscopic surgery, Enhanced Recovery After Surgery

## Abstract

**Objective:**

To enhance postoperative recovery in gynecological malignancies by evaluating the clinical efficacy and safety of Enhanced Recovery After Surgery (ERAS) combined with enteral nutrition.

**Methods:**

This study included 127 patients with gynecological malignancies treated at Zigong Fourth People’s Hospital between January and December 2022. Based on patient preference, 60 were placed in the study group (ERAS + enteral nutrition) and 67 in the control group (traditional care). The two groups were compared on postoperative protein levels, electrolyte balance, and recovery indicators, including the time to first anal exhaust, defecation, and hospital stay duration. Additionally, preoperative thirst, hunger, anxiety scores, postoperative complications, and total hospitalization costs were evaluated.

**Results:**

The study group had higher postoperative total protein (66.59 ± 10.97 g/L) and albumin (43.47 ± 51.27 g/L) than the control group, with a significantly lower incidence of hypoproteinemia (18.33% vs. 50.75%, *p* < 0.05). There were no significant differences in hemoglobin, lymphocyte count, or electrolytes. The study group recovered bowel function earlier than the control group, with significant differences in defecation time (*p* < 0.05). No significant differences were observed in anal exhaust time, hospital stay, complications, or total costs. The study group had better anxiety, thirst, and hunger scores (*p* < 0.05).

**Conclusion:**

ERAS combined with enteral nutrition is safe and effective for gynecological cancer surgery, reducing hypoproteinemia, promoting gastrointestinal recovery, and improving patient experience and psychological well-being.

## Introduction

Gynecological malignant tumors pose a serious threat to women’s physical and mental health. Common types include cervical, ovarian, and endometrial cancer. The most commonly used surgical methods are radical surgery and tumor cell elimination (1). The patient’s postoperative recovery may be affected by factors such as the extensive nature of the surgery, prolonged operation time, significant blood loss, and numerous surgical complications. ERAS is a set of practical and effective treatment and nursing interventions used during the perioperative period. It aims to minimize the stress response and complications in surgical patients, thereby promoting their postoperative recovery (2, 3). Providing appropriate enteral nutrition support during the perioperative period can also enhance the patient’s postoperative recovery, reduce complications, and potentially shorten the hospital stay (4, 5). However, many aspects of applying ERAS in gynecological tumor surgery remain unknown (6). ERAS was first proposed by the Danish surgeon Kehlet (7) and has since been widely implemented. Beyond advocating minimally invasive techniques, ERAS emphasizes the impact of comprehensive perioperative interventions on patient outcomes (8, 9). ERAS integrates evidence-based recommendations to attenuate physiologic and psychologic stress responses to surgery, thereby promoting recovery, reducing postoperative complications, and improving patient outcomes (10). Its overarching goal is to deliver the least harm, the greatest benefit, and the highest quality of care. Successful implementation requires close collaboration across surgery, anesthesia, nursing, nutrition, and psychology (11). Implementation demands active multidisciplinary participation because postoperative recovery is multifactorial and integrative. By synthesizing current evidence, ERAS emphasizes comprehensive pre-, intra-, and postoperative management—such as analgesia, nutritional support, and psychological counseling—to minimize physiologic and psychologic impact. ERAS also underscores patient education and self-care, encouraging active participation in postoperative rehabilitation and stronger self-management. Broader implementation improves patient satisfaction and outcomes and enhances the efficiency and quality of care delivered by clinical teams.

Møller et al. (12) first applied ERAS to gynecologic surgery; the approach was subsequently adopted and disseminated. Studies report that the prevalence of malnutrition among patients with gynecologic malignancies ranges from 62 to 88% (13, 14). Approximately 20% of deaths are attributable to malnutrition (15). In the perioperative setting, malnutrition in patients with gynecologic cancer is associated with higher postoperative complication rates, longer hospital stays, and poorer quality of life (16, 17). This study applied the concept of Enhanced Recovery After Surgery combined with enteral nutrition intervention to patients with gynecological malignant tumors, aiming to improve postoperative recovery and understand the clinical efficacy and safety of this approach. Some results have been achieved and are reported as follows.

## Materials and methods

### The inclusion and exclusion criteria

**Figure 1:**
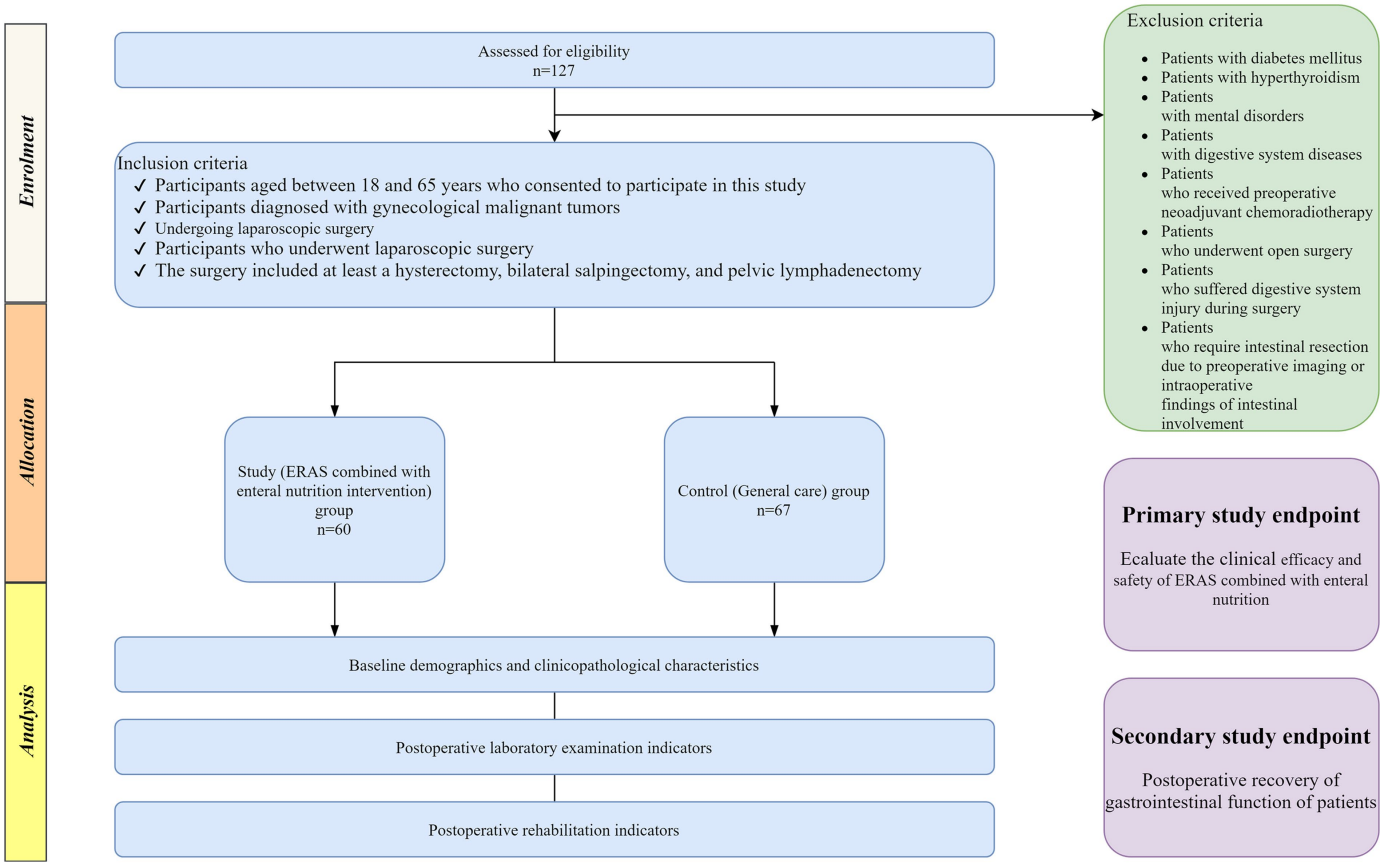
Research process of this paper.

From January to December 2022, our hospital treated 127 patients with gynecological malignant tumors. These patients were selected for this research. Patients were divided into a study group and a control group for a prospective controlled study, based on their preferences. The study group underwent ERAS combined with enteral nutrition intervention, whereas the control group received general nursing care. The study group consisted of 60 cases, and the control group included 67 cases. Inclusion criteria were as follows: (1) Participants aged between 18 and 65 years who consented to participate in this study; (2) Participants diagnosed with gynecological malignant tumors (cervical cancer, endometrial cancer, ovarian cancer); Cervical cancer and endometrial cancer need to be diagnosed through preoperative pathological biopsy. Ovarian cancer patients are diagnosed through preoperative imaging examination, and the diagnosis is supported by postoperative pathological biopsy. (3) Participants who underwent laparoscopic surgery. Surgical treatment shall be carried out within 1 week after clinical diagnosis. (4) The surgery included at least a hysterectomy, bilateral salpingectomy, and pelvic lymphadenectomy. Exclusion criteria were as follows: (1) Patients with diabetes mellitus; (2) Patients with hyperthyroidism; (3) Patients with mental disorders; (4) Patients with digestive system diseases; (5) Patients who received preoperative neoadjuvant chemoradiotherapy; (6) Patients who underwent open surgery; (7) Patients who suffered digestive system injury during surgery; (8) Patients who require intestinal resection due to preoperative imaging or intraoperative findings of intestinal involvement. The implementation of this specific exclusion criterion is due to the significant changes in postoperative recovery pathways and nutritional management caused by intestinal resection, which may also increase the risk of complications such as anastomotic leakage and intestinal obstruction. The hospital ethics committee approved this study, all participating patients provided informed consent, and the work has been reported in line with the STROCSS criteria (18) (Figure 1).

### Methods of intervention

The study group patients underwent an ERAS protocol complemented by enteral nutrition intervention. This primarily involved: (1) Pre-surgery: Two days prior to the operation, patients signed an informed consent for the ERAS protocol combined with enteral nutrition. Additionally, health education was provided to the patients and their families. Patients consumed oral enteral nutrition preparations, formulated by our hospital’s clinical nutrition department, one day before surgery. Specific instructions were to orally mix 45 g of the preparation with 200 mL of warm water, to be taken three times a day, between meals. The primary components of the nutritional preparation included 10 g of whole protein, 10 g of complex protein solid beverage, 10 g of whey protein, and 15 g of maltodextrin. Each bag could provide 180 kcal of energy. Maltodextrin provided 1,598 kJ of energy per 100 g, with 94.0 g of carbohydrates and 70 mg of sodium. It contained no protein or fat. The protein complex solid drink provided 1,641 kJ of energy per 100 g, with 16.0 g of protein, 3.0 g of fat, and 74.0 g of carbohydrates. It also contained 330 mg of sodium, vitamins (300pgRE of A, 2.2ug of D, 4.20 mg of E, 0.45 mg of B1, 0.45 mg of B2, 0.49 mg of B6, 1.00 mg of B12, and 50.0 mg of C), 5.70 mg of niacin, 80 mg of folic acid, 2.00 mg of pantothenic acid, and minerals (100 mg of phosphorus, 320 mg of potassium, 230 mg of calcium, 4.5 mg of iron, and 4.5 mg of zinc). Patients were required to fast for 6 h prior to surgery, with liquid food prohibited 2 h before the procedure. Two hours before surgery, patients were administered a 300 mL 10% glucose solution. (2) Intra-operation: The operating room was maintained at a temperature of 24–36 °C and a humidity of 50.0–60.0%. (3) Anesthesia: Intravenous general anesthesia was employed. (4) Thermoregulation: During the operation, the patient’s body temperature was maintained at 36–37 °C through the use of a heating device for liquid infusion and a warming fan. (5) Infusion Control: Nurses were tasked with monitoring and adjusting the volume and rate of infusion to maintain the patient’s normal blood pressure. (6) Vital Sign Monitoring: The nursing staff closely observed and recorded changes in the patient’s vital signs during the operation, including regular temperature measurements. Individual patient performance was also monitored, with targeted nursing measures implemented as needed. (7) Post-operation: Under the guidance of anesthesiologists, nurses utilized a patient-controlled intravenous analgesia pump for 48 h to alleviate pain. Psychological interventions were also provided during this period to effectively reduce pain sensitivity. (8) Diet: Six hours post-operation, patients were given water. If tolerated, they could consume rice soup or clear porridge. Oral enteral nutrition preparations were reintroduced 12–24 h post-operation and continued for 2–3 days. Specific instructions were to orally mix 45 g of the preparation with 200 mL of warm water, to be taken three times a day, between meals. Normal diet was resumed once bowel sounds normalized. (9) Postoperative Activities: Patients were assisted with ankle pump exercises every 15 min, and encouraged to turn over, take deep breaths, and cough every 2 h. Six hours post-operation, patients were assisted with autonomous in-bed activities. After 24 h, patients were assisted with appropriate ambulation for 0.5–1 h. (10) Drainage Tube Removal: The pelvic drainage tube was scheduled for removal 24–48 h post-operation.

The control group patients were subjected to traditional nursing interventions, primarily comprising: (1) Pre-surgery: Patients’ health status was closely monitored, surgical treatment-related knowledge and precautions were explained, and patients and their families were guided to cooperate with surgery-related nursing and treatment procedures. (2) Pre-surgery Fasting: Patients were strictly required to fast and abstain from drinking for 6 h prior to surgery. An oral sodium phosphate solution was administered for routine bowel preparation from 8 PM to 9 PM the night before surgery. (3) Pre-surgery Enema: A cleansing enema with normal saline was administered on the night before surgery. (4) Intra-operation: The nursing staff strictly adhered to the requirements of anesthesiologists and surgeons, closely monitoring changes in patients’ vital signs and accurately recording them. (5) Post-operation: The nursing staff closely monitored patients for any abnormal conditions, administered analgesic drugs as per the doctor’s advice, and provided postoperative rehabilitation education (Table 1).

**Table 1:** The perioperative nursing plan of the study group and the control group.

Measures to deal with	Study group (*n* = 60)	Control group (*n* = 67)
Preoperative management		
Assessment	The patient’s evaluation of tolerance to surgery and anesthesia	Same as the study group
Preoperative health education	① Education: This was delivered through videos and manuals. ② Communication and Consent: Full communication and informed consent were obtained from patients and their families. Information about ERAS, nutrition interventions, perioperative nursing, postoperative dietary activities, preoperative thirst and hunger scores, and Self-Rating Anxiety Scale (SAS) scores were provided. The preoperative mental and psychological state of the patients was adjusted accordingly. ③ Pre-surgery Lifestyle Changes: Patients were advised to quit smoking and drinking before surgery. Patients were instructed to perform effective expectoration, respiratory function exercises, and lower extremity exercises to prevent venous thrombosis.	Traditional Preoperative Education: ① Perioperative Nursing and Postoperative Dietary Activities: This includes monitoring preoperative thirst and hunger scores, as well as the Self-Rating Anxiety Scale (SAS) score. ② Exercise Guidance: Patients are guided to perform effective expectoration and exercises to prevent venous thrombosis.
Nutritional support	① Pre-surgery Nutrition: Patients consumed oral enteral nutrition preparations, formulated by our hospital’s clinical nutrition department, 1 day before surgery. Usage and Dosage: The specific instructions were to orally mix 45 g of the preparation with 200 mL of warm water, to be taken three times a day, between meals. ② Post-surgery Nutrition: Oral enteral nutrition was reintroduced 12–24 h post-operation and continued for 2–3 days.	None
Bowel preparation	Mechanical Bowel Preparation: ① Pre-surgery Bowel Preparation: An oral sodium phosphate solution was administered from 8 p.m. to 9 p.m. the night before surgery as part of routine bowel preparation. ② Pre-surgery Enema: A cleansing enema with normal saline was performed on the night before surgery.	Same as the study group
Diet	① Pre-surgery Fasting: Patients were advised to avoid prolonged fasting before surgery. ② Pre-surgery Dietary Restrictions: Patients were instructed to abstain from solid food 6 h prior to surgery and from liquid food 2 h before surgery. A 300 mL 10% glucose solution was consumed 2 h before surgery.	One day prior to surgery, patients were placed on a residue-free, semi-liquid diet. Patients were strictly instructed to fast and abstain from drinking for 6 h prior to surgery.
General preoperative preparation	One day prior to surgery, patients are advised to take a routine shower using soap or an antibacterial body wash, clean the navel, prepare the skin, and perform vaginal preparation with povidone-iodine. This preparation is performed the night before surgery and on the morning of surgery.	Same as the study group
Prevention of deep vein thrombosis	① Thrombosis Risk Assessment: The modified Caprini score was utilized to evaluate the thrombosis risk in patients. ② Prophylactic Anticoagulation: Preoperatively, patients were administered a subcutaneous injection of low molecular weight heparin at a dosage of 3,000-6000 IU/d. This was discontinued 24 h prior to surgery.	Same as the study group
Intraoperative nursing management		
Intraoperative heat preservation and thrombosis prevention	① Body Temperature Monitoring: The patient’s body temperature was closely monitored. ② Warming Measures: An insulation blanket was added, the abdominal cavity was rinsed with warm water, and the intravenous fluid was warmed to 36 °C. ③ Thrombosis Prevention: Anti-thrombotic stockings were used in combination with an anti-thrombotic pump.	Routine temperature monitoring
Placement of pipes	① Drainage Tubes: The use of various types of drainage tubes is not generally recommended. However, when necessary, tubes are placed based on the surgical procedure and the patient’s condition, and are removed as soon as possible. ② Indwelling Catheter: The indwelling catheter was removed based on the patient’s condition. In some cases, a few patients could be discharged with the catheter still in place.	① Drainage Tube: The drainage tube was routinely placed and removed based on the patient’s condition. ② Indwelling Catheter: An indwelling catheter was used, and in some cases, a few patients could be discharged with the catheter still in place.
Intraoperative fluid volume management	Implement individualized fluid management to avoid fluid overload or insufficiency	Both strict fluid restriction and open fluid administration protocols were avoided
Postoperative nursing management		
Postoperative pain management	① Postoperative Analgesia: Patient-controlled intravenous analgesia (PCIA) was administered postoperatively. The background dose of sufentanil was set at 2 μg/h, and the PCIA dose was 2 μg per administration. ② Pain Score Assessment: Pain scores were accurately assessed. If the pain score at rest or during activity exceeded 4 points, an anesthesiologist was contacted for management.	Postoperative Analgesia: Patient-controlled intravenous analgesia (PCIA) was administered postoperatively. The background dose of sufentanil was set at 2 μg/h, and the PCIA dose was 2 μg per administration.
Postoperative antiemetic	If necessary, metoclopramide was administered intramuscularly at a dosage of 10 mg per administration.	Same as the study group
Postoperative infusion management	① Postoperative Rehydration: Routine rehydration was performed according to daily requirements, with 1,500 ~ 2000 mL administered on the first postoperative day. ② Second Day Post-operation: On the second day after surgery, the volume of fluid was adjusted based on the patient’s food intake.	Same as the study group
Postoperative nutritional support	① Postoperative Hydration and Nutrition: Following the operation, patients who experienced no nausea or vomiting were given an appropriate amount of water 6 h post-surgery. If tolerated, they could consume rice soup or clear porridge. Oral enteral nutrition preparations were reintroduced 12–24 h post-operation and continued for 2–3 days. ② Postoperative Diet: Six hours post-operation, patients were encouraged to consume high-energy, high-calorie, and high-vitamin liquid/semi-liquid foods, while avoiding milk, soy milk, and sweets to prevent abdominal distension. Eating was encouraged, and actual intake was quantified using graduated measuring cups. ③ Gradual Increase in Food Intake: Food intake was gradually increased based on the patient’s gastrointestinal tolerance. ④ Blood Glucose Control: Blood glucose levels were maintained at less than 11.11 mmol/L.	① Intravenous Nutrition: Intravenous nutrition was continued until the occurrence of anal exsufflation. ② Post-fasting Diet: After 6 h of fasting, a liquid diet was initiated. Following anal exhaust, the diet was gradually transitioned back to normal. ③ Glycemic Control: Blood glucose levels were maintained at less than 11.11 mmol/L.
Recovery of intestinal function	Chew gum for 10–15 min after meals。	None
Postoperative posture and activities	① Bed Positioning: When the patient is conscious, the head of the bed can be elevated to 30° by having the patient lie on their side with a pillow. ② Thrombosis Prevention Exercise: Exercises to prevent deep veting 15 min. ③ Postoperative Mobility: On the first postoperative day, patients were required to walk more than 4 times, with a cumulative out-of-bed time exceeding 2 h. ④ Activity Increase: Patients were encouraged to gradually increase their activity levels.	① Postoperative Positioning: After the operation, patients were returned to the ward and placed in a semi-reclining position following 6 h of lying flat with a low pillow. ② Postoperative Day: Bed activities were encouraged. ③ First Day Post-operation: On the first day after surgery, patients were encouraged to ambulate 3–5 times, with each session lasting 15 min. ④ Activity Increase: Patients were encouraged to gradually increase their activity levels.
Postoperative prevention of deep vein thrombosis	① Postoperative Anticoagulation: Low molecular weight heparin was administered on the first day after surgery. For malignant tumor surgeries, this treatment could continue for up to 28 days. ② Thrombosis Prevention: Sequential compression anti-thrombotic devices were used for patients in bed at the district hospital. ③ Anti-thrombotic Stockings: Patients were instructed on the correct usage of anti-thrombotic elastic stockings.	Same as the study group
Discharge criteria	① Diet Resumption: The patient resumed a solid diet without the need for intravenous infusion. ② Medical Intervention: Medical interventions were carried out without the need for special hospitalization. ③ Independent Activities: The patient was able to carry out certain activities independently, without assistance. ④ Discharge: The patient agreed to be discharged.	Same as the study group

### Data collection

Preoperative thirst score, preoperative hunger score (19): Visual analog combined with numerical scoring method is used; the thirst and hunger of the two groups are assessed, and the degree of thirst and hunger is quantitatively assessed according to a scale of 0~10. This scale is a 10 cm horizontal straight line, composed of 11 points from 0~10, where 0 means no thirst/hunger, 10 means the most thirsty/hungry, and the higher the score, the higher the degree of thirst/hunger. Among them, 0 points mean no thirst/hunger; 1~3 points mean mild, slightly thirsty/hungry; 4~6 points mean moderate, obvious thirst/hunger but tolerable; 7~10 points mean severe, extreme thirst/hunger, intolerable or showing signs of hypoglycemia/dehydration; patients are scored within 1 h before surgery.

Postoperative first anal exhaust time, postoperative first bowel movement time, postoperative hospital stay, total hospital cost. Total hospital costs were defined as the sum of all direct medical costs incurred during the index hospitalization, including fees for surgery and anesthesia; room and board; medications; disposable medical supplies; and laboratory tests and imaging studies. Cost data were extracted from the hospital financial management database. Postoperative hemoglobin, lymphocyte count, total protein, albumin, blood potassium, blood sodium, blood chloride: indicators of laboratory test results on the 3rd–5th day after surgery.

Self-Rating Anxiety Scale (SAS) score (20): there are 20 items in total, each with a four-level score; 1 means none or occasional, 2 means sometimes, 3 means often, 4 means always. Patients, based on the actual situation in the past week, tick “√” under the appropriate score in the score column 1~4; the main statistical indicator of “SAS” is the total score; after the patient’s self-evaluation is over, the scores of the 20 items are added to get the total score, then multiplied by 1.25 to get the integer part, which is the standard score; the higher the standard score, the more severe the symptoms. Patients are scored within 1 h before surgery.

Postoperative complications: include postoperative infection, anemia, hypoproteinemia, electrolyte imbalance, thrombosis.

### Statistical methods

Statistical analysis was conducted using SPSS software (version 25.0). Measurement data conforming to normal distribution are denoted by mean ± standard ($\bar{x}\pm s$). An independent sample t-test is used for comparing two groups, while variance analysis is employed for multiple group comparisons. For measurement data not following a normal distribution, it is represented by Median (interquartile range) *M*(P_25_, P_75_). Both two-group and multi-group comparisons utilize non-parametric tests. Count data are denoted by examples or percentages *n* (%), with both two-group and multi-group comparisons conducted using χ^2^ test analysis. A *p*-value of less than 0.05 is indicative of statistical significance.

## Result

### Comparison of general data between the study group and the control group

The comparison of baseline data such as age, BMI, preoperative blood laboratory test indicators, tumor type composition ratio, intraoperative blood loss, etc. of the two groups of patients, the difference has no statistical significance (*p* > 0.05) (Table 2).

**Table 2:** Comparison of baseline data between the study group and the control group (plus menopause).

	Study group (*n* = 60)	Control group (*n* = 67)	*t*/*χ^2^*	*p*
Age (y)	54.43 ± 9.80	53.43 ± 7.73	0.632	0.530
BMI (kg/m^2^)	24.08 ± 3.60	24.74 ± 3.45	−1.035	0.303
Menopause (*n* %)				
Yes	31 (51.7%)	28 (41.8%)	1.241	0.265
No	29 (48.3%)	39 (58.2)		
Number of pregnancies	3.42 ± 1.46	3.36 ± 1.30	0.238	0.812
Order of birth	1.47 ± 0.50	1.48 ± 0.50	−0.122	0.903
Preoperative hemoglobin (g/L)	121.40 ± 13.52	122.64 ± 12.91	−0.529	0.598
Preoperative LYW (10^9^/L)	1.58 ± 0.74	1.49 ± 0.57	0.751	0.454
Preoperative total protein (g/L)	73.48 ± 7.16	72.84 ± 7.84	0.477	0.634
Preoperative albumin (g/L)	42.76 ± 4.81	42.58 ± 3.67	0.244	0.808
Preoperative potassium (mmol/L)	4.42 ± 4.59	3.80 ± 0.34	1.109	0.270
Preoperative serum sodium (mmol/L)	142.35 ± 2.48	142.12 ± 2.50	0.517	0.606
Preoperative blood chlorine (mmol/L)	104.74 ± 5.07	104.27 ± 1.97	0.700	0.485
Intraoperative blood loss (ml)	222.50 ± 181.20	292.99 ± 279.70	−1.664	0.099
Type of tumor (*n* %)				
Cervical cancer	30 (50.0%)	25 (37.4%)		
Endometrial Cancer	15 (25.0%)	28 (41.8%)	4.046	0.132
Ovarian cancer	15 (25.0%)	14 (20.9%)		

### Comparison of postoperative laboratory examination indexes between the study group and the control group

The postoperative total protein (66.59 ± 10.97 g/L) and albumin (43.47 ± 51.27 g/L) of the research group are both higher than the control group, and the difference is statistically significant (*p* < 0.05). The incidence of hypoproteinemia in the research group is 18.33%, which is lower than the 50.75% in the control group (*p* < 0.05). There is no statistical difference in postoperative hemoglobin, lymphocyte count, blood potassium, blood sodium, and blood chlorine between the two groups of patients (*p* > 0.05).

### Comparison of postoperative rehabilitation indexes and economic indexes between the study group and the control group

The research group showed a statistically significant earlier recovery of defecation post-surgery compared to the control group (*p* < 0.05). No statistical difference was observed between the two patient groups in terms of time for exhaust recovery, length of postoperative hospital stay, postoperative complications, and total hospitalization cost (*p* > 0.05).

### Comparison of self-rating anxiety scale score, preoperative thirst score and preoperative hunger score between the study group and the control group

The research group demonstrated statistically significant improvements over the control group in terms of perioperative anxiety self-rating scale scores, preoperative thirst scores, and preoperative hunger scores (*p* < 0.05) (Table 3).

**Table 3:** Comparison of clinical data between the study group and the control group.

	Study group (*n* = 60)	Control group (*n* = 67)	*t*	*p*
Postoperative hemoglobin (g/L)	106.88 ± 15.27	106.43 ± 16.45	0.159	0.874
Postoperative LYW (10^9^/L)	1.10 ± 0.45	1.18 ± 0.75	−0.643	0.521
Postoperative total protein (g/L)	66.59 ± 10.97	55.45 ± 9.43	6.151	0
Postoperative albumin (g/L)	43.47 ± 51.27	29.91 ± 5.34	2.153	0.033
Hypoproteinemia (*n* %)	11/60 (18.33%)	34/67 (50.75%)	14.436	0
Postoperative potassium (mmol/L)	3.78 ± 0.40	3.75 ± 0.31	0.414	0.680
Postoperative serum sodium (mmol/L)	140.49 ± 2.27	139.98 ± 2.52	1.200	0.232
Postoperative blood chlorine (mmol/L)	103.18 ± 2.82	103.42 ± 2.64	−0.500	0.618
The first postoperative anal exsufflation time (d)	2.43 ± 1.23	2.73 ± 0.96	−1.531	0.128
First postoperative anal defecation time (d)	4.37 ± 1.74	5.03 ± 1.80	−2.107	0.037
Length of postoperative hospital stay (d)	7.98 ± 3.63	8.24 ± 3.22	−0.420	0.675
Preoperative thirst score	2.7 ± 1.27	4.94 ± 1.10	−10.672	0
Preoperative hunger score	3.07 ± 0.95	5.10 ± 1.06	−11.330	0
SAS score	26.37 ± 5.49	40.76 ± 9.84	−10.015	0
Postoperative complications^a^ (*n* %)	35 (58.33%)	38 (56.72%)	0.034	0.854
Total cost of hospitalization	2.80 ± 0.69	3.15 ± 0.84	−2.54	0.012

^a^Postoperative complications: In the experimental group, there were 35 instances of postoperative complications, which included 13 infections, 30 cases of anemia, 11 instances of hypoproteinemia, and 11 cases of hypokalemia. The control group reported 38 instances of postoperative complications, comprising 16 infections, 37 cases of anemia, 34 instances of hypoproteinemia, and 13 cases of hypokalemia.

## Discussion

Patients with malignant gynecological tumors often require extensive surgery, including radical resection. The surgery is invasive, involves a lengthy operation time, may result in significant bleeding, and is associated with a slow postoperative recovery. Post-surgery, the body typically experiences significant stress and a negative nitrogen balance, limiting the utilization of exogenous nutrients. Therefore, postoperative nutritional support is crucial. Concurrently, malnutrition in patients with gynecological tumors during the perioperative period can lead to an increased incidence of postoperative complications, extended hospital stays, and a diminished quality of life. Gynecologic cancers—especially advanced ovarian cancer—can involve the gastrointestinal tract and may require colorectal procedures (e.g., rectal or sigmoid resection). To minimize the risk of postoperative bowel obstruction and anastomotic leakage, we excluded patients who required intestinal surgery. All enrolled patients had intact gastrointestinal anatomy, and the index operation did not materially alter the digestive tract. Therefore, perioperative administration of oral enteral nutrition formulas to enhance nutritional support was feasible. This nutritional approach not only aids in accelerating patient recovery and reducing the incidence of complications like hypoproteinemia, but it can also decrease fluid volume during the perioperative period, lessen patient burden, and enhance the patient’s medical experience. Additionally, enteral nutrition agents can promote post-surgery gastrointestinal recovery, enhance the patient’s medical experience, and facilitate a quicker return to normal life and work. Thus, for patients with malignant gynecological tumors, the judicious selection and use of enteral nutrition agents are vital, as they can assist patients in better managing surgical challenges and improving overall treatment outcomes and quality of life. A prospective randomized controlled study by Steed et al. (21) demonstrated that, for patients undergoing major gynecological abdominal surgery, early postoperative oral feeding can reduce hospital stay compared to the control group, with no significant difference in the incidence of complications such as vomiting and intestinal obstruction. Heyland et al. (22) conducted a meta-analysis examining the impact of total parenteral nutrition support versus a regular diet plus intravenous glucose infusion on surgical patients. The results indicated that total parenteral nutrition support can significantly reduce postoperative infection complications in malnourished patients, but it does not affect mortality. A randomized controlled study involving 338 gynecological tumor laparotomy patients, of which 112 cases (33%) received perioperative immune regulation dietary supplement treatment, found that the addition of dietary supplements can effectively reduce wound complications in these patients (23).

In our study, we found that that the research group had higher postoperative total protein and albumin levels than the control group, a difference that was statistically significant (*p* < 0.05). Additionally, the incidence of hypoproteinemia was significantly lower in the research group (*p* < 0.05). The research group exhibited a significantly earlier recovery of defecation post-surgery compared to the control group (*p* < 0.05). No significant difference was observed in postoperative hospital stay duration, postoperative complications, and total hospitalization costs. Consequently, the researcher posits that the combination of ERAS and oral enteral nutrition agents is safe and feasible, can effectively lower the incidence of hypoproteinemia, enhance post-surgery digestive tract function recovery, and will not significantly escalate the patient’s medical economic burden. Yeung SE (5) discovered that patients in the ERAS group could expedite postoperative recovery and reduce hospital stay duration by increasing protein intake through oral nutritional supplements. Results from a large-scale cohort study indicate that incorporating oral carbohydrates into the ERAS program can significantly enhance patient clinical outcomes (24). The meta-analysis of Bisch (25) concluded that the ERAS program reduces hospital stay duration, complications, and costs, without increasing the readmission rate or mortality associated with gynecological tumor surgery. This supports the adoption of ERAS as the nursing standard for gynecological tumors.

ERAS not only offers clear health economic benefits (26) but also yields high patient satisfaction (27). Systematic implementation of the ERAS gynecological cancer guidelines across the healthcare system can enhance patient prognosis and conserve resources. Bisch et al. (28), through research involving 152 gynecological cancer patients who did not implement ERAS and 367 who did, confirmed that systematic implementation of ERAS gynecological cancer perioperative management measures across the medical system can enhance patient prognosis and decrease hospitalization costs. The study compared the anxiety self-rating scale scores, preoperative thirst scores, and preoperative hunger scores of the two groups, finding that the research group significantly outperformed the control group (*p* < 0.05). This indicates that the combination of ERAS and oral enteral nutrition agents can significantly enhance the patient’s medical experience, alleviate the patient’s mental and psychological burden, and boost patient satisfaction.

We implemented an ERAS protocol incorporating enteral nutrition without additional personnel or resources, relying on collaboration within the existing multidisciplinary team (gynecologists, anesthesiologists, nurses, and clinical dietitians). This study has several limitations. First, allocation was preference-based rather than randomized. Second, the single-center design and relatively small sample size may limit statistical power and generalizability. Moreover, although we described the components and intensity of the nutritional intervention, caloric and protein targets were not standardized and adherence to oral nutritional supplements was not objectively monitored. Therefore, these findings require confirmation in adequately powered randomized controlled trials.

## Conclusion

The application of ERAS in conjunction with oral enteral nutrition agents in the surgical treatment of patients with malignant gynecological tumors is safe and feasible. It can notably decrease the incidence of hypoproteinemia, foster post-surgery digestive tract function recovery, enhance the patient’s medical experience, and alleviate the patient’s mental and psychological burden.

## Data Availability

The original contributions presented in the study are included in the article/supplementary material, further inquiries can be directed to the corresponding authors.
